# The Role of Autophagy and NLRP3 Inflammasome in Liver Fibrosis

**DOI:** 10.1155/2020/7269150

**Published:** 2020-07-11

**Authors:** Ye Tao, Ningning Wang, Tianming Qiu, Xiance Sun

**Affiliations:** ^1^Occupational and Environmental Health Department, Dalian Medical University, 9 Lvshun South Road, Dalian 116044, China; ^2^Nutrition and Food Hygiene, Dalian Medical University, 9 Lvshun South Road, Dalian 116044, China; ^3^Global Health Research Center, Dalian Medical University, 9 Lvshun South Road, Dalian 116044, China

## Abstract

Liver fibrosis is an intrinsic repair process of chronic injury with excessive deposition of extracellular matrix. As an early stage of various liver diseases, liver fibrosis is a reversible pathological process. Therefore, if not being controlled in time, liver fibrosis will evolve into cirrhosis, liver failure, and liver cancer. It has been demonstrated that hepatic stellate cells (HSCs) play a crucial role in the formation of liver fibrosis. In particular, the activation of HSCs is a key step for liver fibrosis. Recent researches have suggested that autophagy and inflammasome have biological effect on HSC activation. Herein, we review current studies about the impact of autophagy and NOD-like receptors containing pyrin domain 3 (NLRP3) inflammasome on liver fibrosis and the underlying mechanisms.

## 1. Introduction

Liver fibrosis refers to a compensatory response of liver inflammation and damage caused by various pathogenic factors. Liver fibrosis has high morbidity and high mortality worldwide and is a common characteristic shared by many liver injuries, such as hepatitis B, autoimmune hepatitis, alcoholic liver disease, nonalcoholic steatohepatitis, and hepatocellular carcinoma [1]. After the emergence of steatosis and steatohepatitis, liver fibrosis will shape up gradually without timely cure. Liver fibrosis is characterized by excessive deposition of extracellular matrix (ECM), including collagen, glycoproteins, and proteoglycans, and severe fibrosis can lead to cirrhosis, portal hypertension, and even cancer [2]. It is well known that the activation of hepatic stellate cells (HSCs) plays a crucial role in the process of liver fibrosis. Upon stimulation by liver damage, the quiescent HSCs are activated and converted into myofibroblasts, which release a large amount of ECM, form scar tissues, and eventually lead to liver fibrosis [3, 4].

Autophagy is a process of degrading excess or damaged organelles or other components in the cell with the participation of lysosomes [5]. Autophagy occurs in all kinds of nucleated cells and is essential for cell survival, differentiation, growth, and homeostasis [6]. Therefore, dysregulated autophagy is involved in the development of various diseases, such as cancer, neurodegeneration, and diabetes [7]. It is no doubt that, as a physiological function of a cell, autophagy can also regulate the activity of HSCs. In the process of liver fibrosis, autophagy works as a double-edged sword. On the one hand, the upregulated autophagy can activate HSCs, promoting the development of fibrosis; on the other hand, excessive autophagy may inhibit the development of fibrosis [8, 9].

Inflammasome is a macromolecular protein complex, which is assembled by intracellular pattern recognition receptors. As an important component of the innate immunity, inflammasome promotes the maturation and secretion of proinflammatory factors [10]. Among all known inflammasomes, the NOD-like receptor family is the most characteristic, of which the NOD-like receptors containing pyrin domain 3 (NLRP3) inflammasome is a classic member [11]. Some researches found that NLRP3 inflammasome takes part in the development of sterile inflammation, which is induced by sterile cell death in the condition of mechanical trauma, ischemia, stress, and environmental cues [12]. In addition, sterile inflammation is regarded as a significant factor for fibrosis [13]. Thus, the NLRP3 inflammasome is associated with liver fibrosis to a great extent.

To sum up, autophagy and NLRP3 inflammasome are significant for liver fibrosis. The main purpose of this review is to systematically summarize the effects of autophagy and NLRP3 inflammasome on liver fibrosis and the underlying mechanisms.

## 2. The Overview of Liver Fibrosis

Liver fibrosis is a pathological state in which the ECM is secreted and deposited largely in response to liver injury, which disrupts the normal structure and function of the liver. There are many causes of liver fibrosis, such as viral or parasitic infections, cholestasis, metabolic diseases, and excessive intake of alcohol chronically [14]. Furthermore, these pathogenic factors induce liver fibrosis ultimately through inflammation and oxidative stress damage [15]. Age, alcohol intake, and gender are considered to be the three major factors affecting liver fibrosis, and about 33% of patients with liver fibrosis worldwide will progress to cirrhosis, if not being treated in time [16]. What is worse, about 85% of patients with cirrhosis will be diagnosed with hepatocellular carcinoma [17]. Liver fibrosis is regarded as a common pathological basis of numerous liver diseases. For instance, approximately 57% of patients with nonalcoholic steatohepatitis (NASH) are suffering from different degrees of liver fibrosis [18]. Liver fibrosis is a reversible pathological state, so the prevention and treatment of liver fibrosis are of great significance for prevention of various liver diseases.

### 2.1. Potential Pathogenesis of Liver Fibrosis

#### 2.1.1. The Role of Hepatic Stellate Cell Activation in Liver Fibrosis

Hepatic stellate cells (HSCs), known as fat-storing cells (FSCs), are mainly distributed in the hepatic sinusoidal space. The quiescent HSCs are rich in vitamin A, accounting for 8% to 14% of the total amount in hepatocytes. When stimulated by profibrotic factors, the quiescent HSCs are activated and transformed into myofibroblasts, storing and secreting large amounts of ECM [19–21]. ECM is mainly composed of *α* smooth muscle actin (*α*-SMA) and collagen, and as we mentioned before, excessive deposition of ECM could destroy the normal liver structure. At the same time, the activated HSCs could further proliferate and migrate, aggravating the process of liver fibrosis [22]. Except for ECM, HSCs also express matrix metalloproteinases (MMPs) and tissue inhibitors of metalloproteinases (TIMPs), which play important roles in the progression of liver fibrosis [23]. MMPs are defined as a group of extracellular endopeptidases consisting of 25 members [24, 25]. The family of MMPs is responsible for reversal and degradation of all components of ECM. Among the members of MMPs, some have the capacity to inhibit fibrosis, such as MMP-1, MMP-8, and MMP-13. Their upregulation can alleviate liver fibrosis and promote the proliferation of hepatocyte [26]. On the contrary, some MMP members such as MMP-12 and MMP-19 are fibrogenic. TIMPs are the endogenous inhibitors of MMPs. TIMPs are composed of TIMP-1, TIMP-2, TIMP-3, and TIMP-4, which bind to MMPs with 1 : 1 noncovalent stoichiometry [27]. In general, the unbalanced expression of MMPs and TIMPs after activation of HSCs may be an important contributor to the liver fibrosis progression. Some signals have been found about autophagy and NLRP3 inflammasome involved in HSC activation, such as the autophagic-CTSB-inflammasome axis [28] and P2X7R-NLRP3 inflammasome pathway [29]. Activation of HSCs is a crucial step in the initiation of liver fibrosis. There are numerous causes of HSC activation, among which inflammation and oxidative stress are two important aspects.

#### 2.1.2. The Role of Oxidative Stress in Liver Fibrosis

**Figure 1:**
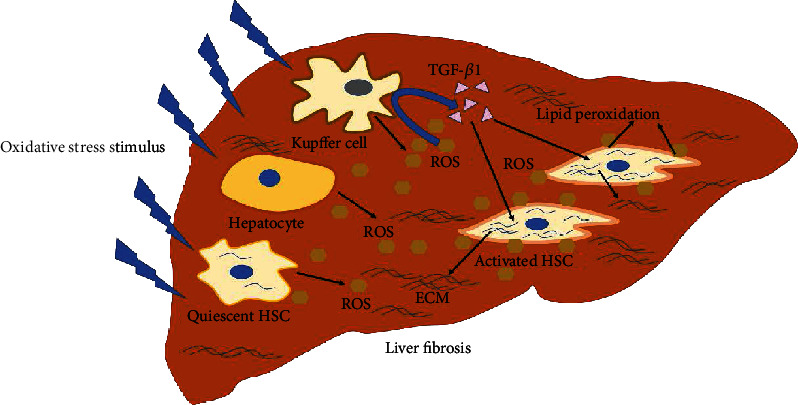
Oxidative stress plays an important role in HSC activation and liver fibrosis. In the liver, under impact of harmful stimulus, oxidative stress is induced and hepatocytes, Kupffer cells, and HSCs will produce mass of ROS. On the one hand, the ROS bond with cytomembrane of HSCs and cause its lipid peroxidation, leading to HSC activation. On the other hand, ROS will stimulate Kupffer cells to produce and release TGF-*β*1 in turn, which can induce HSC activation admittedly.

Oxidation-antioxidant imbalance occurs when reactive oxygen species and free radicals accumulate excessively beyond the capacity of the antioxidant system and ultimately leads to oxidative stress [30]. Free radicals are by-products of cellular metabolism with high activity and reactivity, due to the unpaired electrons on the outermost layer. Free radicals are generated in various parts of the body by several stimulations, such as long-term stress, disease, and ultraviolet exposure [31, 32]. Reactive oxygen species (ROS) are chemically reactive molecules produced during oxygen-related reactions. When the liver is attacked by harmful stimulations, hepatocytes, Kupffer cells, and HSCs themselves will produce large amounts of ROS [33, 34], which react directly with intracellular lipids, proteins, and DNA, furtherly causing cell damage and death. Moreover, ROS can also activate HSCs through lipid peroxidation on the cell surface, leading to liver fibrosis. As found by Casini et al., followed by N-formyl-Met-Leu-phenylalanine (FMLP) stimulation, neutrophil-produced ROS induced HSC activation in the coculture model [35]. In addition, during oxidative stress, ROS can induce HSC activation by stimulating Kupffer cells and inflammatory cells to secrete profibrogenic factors, including transcription growth factor, cytokines, and chemokines. For instance, TGF-*β*1 is an acknowledged inducer for activating HSCs, which is released from Kupffer cells by excessive ROS [36]. Collectively, we can see that excessive oxidative stress is an important factor driving liver injury and fibrosis. Therefore, it is a significant strategy to attenuate oxidative stress for the treatment of liver fibrosis (Figure 1).

#### 2.1.3. The Role of Inflammation in Liver Fibrosis

**Figure 2:**
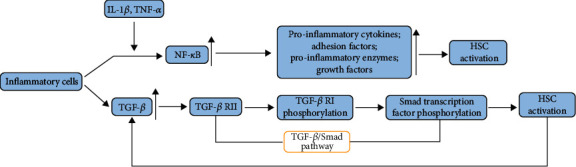
Inflammatory cells are involved in HSC activation. As shown in the picture, NF-*κ*B pathway inflammatory cells of the liver can be activated through cytokines, such as IL-1*β* and TNF-*α*. After that, various profibrotic factors will be released and activate HSCs. Besides, the TGF-*β*, produced by inflammatory cells, can also activate HSCs via the TGF-*β*/Smad pathway.

Inflammation is a defensive reaction to external invasion or damage, accompanied by congestion, exudation, and inflammatory cell infiltration [37]. When the liver suffers sustained damage, inflammatory cells and parenchymal cells secrete large amounts of cytokines and chemokines, such as interleukin-1*β* (IL-1*β*), tumor necrosis factor-*α* (TNF-*α*), transforming growth factor-*β* (TGF-*β*), and IL-6, which further cause the aggregation of inflammatory cells and amplify inflammatory response. Among these cytokines, IL-1*β* and TGF-*β* are closely related to liver fibrosis due to their capacity to activate HSCs [38, 39]. Moreover, TGF-*β* has been demonstrated to activate HSCs through the TGF-*β*/Smad pathway. In turn, the activated HSCs express TGF-*β*, thereby forming the inflammatory cascade [40]. Besides, inflammation-induced activation of the NF-*κ*B signaling pathway is also associated with liver fibrosis. Specifically, after NF-*κ*B is activated by proinflammatory factors, such as IL-1*β* and TNF-*α*, the I*κ*B proteins are degraded as the I*κ*B kinase complexes are activated. Then, the I*κ*B proteins translocate to the nucleus and bind to the DNA binding sites, promoting fibrosis by regulating its targeting proinflammatory cytokines (IL-1, IL-2, IL-6, and TNF-*α*), adhesion factors (ICAM, VCAM, and E-selectin), proinflammatory enzymes (COX-2, iNOS), and growth factors (TGF-*β*) [41–43]. Simultaneously, activation of the NF-*κ*B signaling pathway will further promote the accumulation of proinflammatory chemokines, leading to amplification of the NF-*κ*B signaling pathway furtherly, along with HSC activation to a greater extent [38] (Figure 2).

## 3. The Role of NLRP3 Inflammasome and Autophagy in Liver Fibrosis

### 3.1. The Overview of NLRP3 Inflammasome

Inflammasome, as a biological macromolecule, is a component of innate immunity and plays a significant role in the host defense system and inflammatory signaling platform. It is responsible for detecting the damage factors during infection and tissue damage and activating the inflammatory response. NLRP3 inflammasome, composed of a nucleotide-binding oligomerization domain-like receptor 3 (NLRP3), an apoptosis-associated spot-like protein containing a caspase recruitment domain (ASC), and a serine protease caspase-1 [44], has remarkable effects on innate immunity and various inflammatory diseases, such as type 2 diabetes, atherosclerosis, and neurodegenerative diseases. In addition, neutrophils are involved in the sterile inflammatory response, and the NLRP3 inflammasome pathway plays an important role in the function of neutrophils [45]. Thus, it can be seen that NLRP3 inflammasome activation is associated with liver inflammation and fibrosis. The process of NLRP3 inflammasome activation is composed of two parts: primer phase (signal 1) and trigger phase (signal 2). Signal 1 is induced by LPS, and signal 2 is composed of damage-associated molecular patterns (DAMPs) and pathogen-associated molecular patterns (PAMPs), including intracellular ionic fluxes (Ca^2+^ influx and K^+^ efflux), ROS, and lysosomal damage. It has been reported that low concentration of K^+^ acts at the upstream signal of NLRP3 activation, and K^+^ efflux can be induced by ATP. In addition, mitochondrial Ca^2+^ overload may cause mitochondrial dysfunction and associated ROS generation [46].

### 3.2. The Activation of NLRP3 Inflammasome Promotes HSC Activation through Inflammatory Response

**Figure 3:**
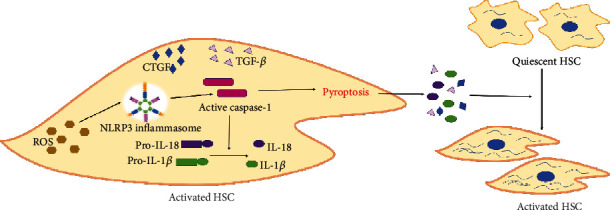
The activation of NLRP3 inflammasome is a key step of HSC activation. Lots of studies have testified that ROS is an activator of NLRP3 inflammasome. In HSCs, NLRP3 inflammasomes are activated by ROS and the procaspase-1 is transformed into activated caspase-1. Then, pro-IL-18 and pro-IL-1*β* were activated into mature IL-18 and IL-1*β*. Moreover, caspase-1 can also cause pyroptosis of HSCs. After pyroptosis, cytomembrane is ruptured and the cytoplasmic contents will be released into extracellular environment, such as IL-18, IL-1*β*, TGF-*β*, and CTGF, leading to HSC activation.

In the murine model of NASH, activation of NLRP3 inflammasome was indispensable for fibrotic response [44]. Moreover, several studies have shown that all components of NLRP3 inflammasome are present in HSCs, which regulate a variety of functions of HSCs and are associated with HSC activation [47]. After activation of NLRP3 inflammasome, pro-IL-18 and pro-IL-1*β* are cleaved by caspase-1 into matured IL-18 and IL-1*β*. As two downstream cytokines of NLRP3 inflammasome, IL-18 and IL-1*β* can promote the transformation of HSCs into mechanocytes [48, 49]. In addition, pyroptosis can be induced by caspase-1 followed by the activation of NLRP3 inflammasome in cells. Afterwards, the cytoplasmic contents, including IL-1*β*, IL-18, TGF-*β*, and connective tissue growth factor (CTGF), will be released into the extracellular environment, facilitating the development of liver fibrosis. However, fibrosis will be mitigated when hepatic inflammation was alleviated by the NLRP3 inhibitor [50, 51]. Besides, increased ROS in HSCs may also lead to cell activation via activating NLRP3 inflammasomes [52]. Therefore, NLRP3 inflammasomes play a potential role in the activation of HSCs and the development of liver fibrosis, but there are few relevant studies so far (Figure 3).

### 3.3. The Overview of Autophagy

Autophagy is the process of degrading harmful components in cells including misfolded proteins, damaged organelles, and excessive lipids, to maintain the cellular components and homeostasis [53]. Currently, three types of autophagy have been identified, including macroautophagy, microautophagy, and chaperone-mediated autophagy, among which macroautophagy is the most common form. This review will focus on macroautophagy (hereinafter referred to as autophagy). The general process of autophagy is to gradually encapsulate cytoplasmic components into vesicles of double-layer membranes to form autophagosomes, which then bind to lysosomes and degrade in lysosomes [54]. Autophagy is regulated by numerous autophagy-related genes (ATG), such as Beclin-1, ATG4, Light Chain 3 (LC3, ATG8), and p62. Of note, when autophagy is initiated, LC3I is lipidated to LC3II, which is involved in the formation of autophagosomes. The adaptor protein sequestosome 1 (p62) serves as substrates to LC3II and is degraded along with cargo. Therefore, LC3II and p62 are regarded as biomarkers of autophagic flux [55, 56]. In general, autophagy maintains at a basal level to facilitate cell survival. However, the autophagy level rises in circumstances of inflammation, oxidative stress, and nutrient deprivation. In turn, excessive autophagy will lead to autophagic cell death and is involved in infectious, inflammatory, and liver diseases [54, 57, 58].

### 3.4. Autophagy Upregulation Facilitates HSC Activation

Autophagy is a crucial manner for cells to adapt to various stress states. During the process of liver fibrosis, the involvement of autophagy in the mechanism of HSC activation has attracted increasing attention [59]. In the study of Li et al., on the one hand, autophagy could activate HSCs, increase the release of ECM, and aggravate the development of fibrosis; on the other hand, autophagy could also negatively modulate the activation of HSCs [58]. It has been demonstrated that autophagy can provide energy for HSC activation. A major feature of HSC activation is the release of lipid droplets containing retinol (vitamin A) and triglycerides, while autophagy can degrade lipid droplets in HSCs and hydrolyze the retinol into free fatty acids, which are further oxidized by mitochondria to produce ATP and provide energy for cell activation [60–62]. Inhibition of autophagy will reduce the degradation of lipid droplets, thereby preventing the activation of HSCs. Except that, during the autophagic process, lysosome-contained cathepsin B, an activator of NLRP3 inflammasome, will be released into cytoplasm [63], which can lead to NLRP3 inflammasome activation and final HSC activation.

### 3.5. Excessive Autophagy Inhibits Liver Fibrosis

**Figure 4:**
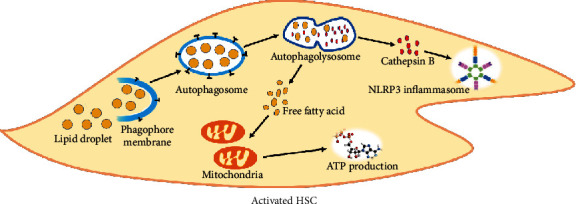
Autophagy participate in HSC activation. Autophagy is the normal physiological function of cells and maintain the balance of energy and substances. However, abnormal autophagy can also induce HSC activation. The consumption of lipid droplets is a character of HSC activation. Therefore, lipid droplets can be resolved into free fatty acid though autophagy. Then, mitochondria will produce ATP by consuming free fatty acid, providing energy for HSC activation. In addition, cathepsin B can be released into cytoplasm due to destruction of the lysosomal membrane. Cathepsin B has been demonstrated to be an inducer of NLRP3 inflammasome activation. So, this is another approach of activating HSCs by autophagy.

On the contrary, several studies have suggested that autophagy can inhibit the progression of liver fibrosis. Lodder et al. have found that autophagy inhibits Kupffer cells from secreting cytokines such as IL-1*β*, thereby preventing HSC activation [64]. Then, Chen et al. have demonstrated that cells were damaged by excessive autophagy, resulting in HSC aging, followed by declining activity and reduced ECM secretion [9]. Therefore, autophagy may play a dual role in the process of liver fibrosis, and more evidence is needed (Figure 4).

## 4. Autophagy and NLRP3 Inflammasome Activate HSCs via Hedgehog Signaling

Hedgehog (Hh) is a developmental regulator discovered firstly in modulating embryonic morphogenesis [65]. Besides, Hh signaling is also involved in wound healing in adult tissues [66]. The Hh signaling is activated when Hh ligands interact with receptor Patch (Ptch) on the membrane of Hh-responsive cells. After that, the nuclear transcription of the glioma-associated oncogene transcription factor (Gli) family is motivated, which regulates the expression of Hh target genes associated with cell growth and transformation [67]. In the liver, hepatocytes are the main producers of Hh ligands. After hepatocyte death, a mass of Hh ligands is released and stimulates Hh-responsive cells through liver sinusoids and bile canaliculi. It has been demonstrated that HSCs are Hh responsive and will be transformed into fibrogenic myofibroblasts (MFs). However, HSC activation can be inhibited by interfering Hh signaling [67, 68]. It is well-known that excessive autophagy and NLRP3 inflammasome activation can induce autophagic cell death and pyroptosis, respectively [69]. Therefore, if hepatocytes suffer autophagic cell death and pyroptosis, Hh ligands can be released and HSCs may be activated by Hh signaling, leading to liver fibrosis at last.

## 5. Conclusion and Future Perspectives

In summary, the process of liver fibrosis includes the activation of HSCs and the deposition of ECM. Moreover, autophagy and NLRP3 inflammasome have significant effects on the process of liver fibrosis. As the main downstream signaling of NLRP3 inflammasome, the IL-1 family pathway is a vital mediator of liver injury and fibrogenesis. However, the link between them is complicated, and there have been few studies about the combined effects of autophagy and NLRP3 inflammasome on HSC activation and liver fibrosis. In addition, the regulation of autophagy and NLRP3 inflammasome might be identified as the target for the treatment of liver fibrosis, and further clinical trials are required to verify it.
